# Novel pathogenic *CTLA4* missense variant associated with autoimmune enteropathy and systemic immune dysregulation

**DOI:** 10.3389/fimmu.2026.1855020

**Published:** 2026-07-27

**Authors:** Danping Zheng, Chunyang Tian, Xiaoqi Ye, Ziyin Ye, Gayatree Mohapatra, Haiyuan Ma, Baili Chen, Zhirong Zeng, Shenghong Zhang, Taofeng Jiang, Yao He, Minhu Chen

**Affiliations:** 1 Department of Gastroenterology, The First Affiliated Hospital, Sun Yat-sen University, Guangzhou, China; 2 Department of Gastroenterology, Huiya Hospital of The First Affiliated Hospital, Sun Yat-Sen University, Huizhou, China; 3 Department of Pathology, The First Affiliated Hospital, Sun Yat-sen University, Guangzhou, China; 4 Division of Microbiome and Cancer, German Cancer Research Center (DKFZ), Heidelberg, Germany; 5 Department of Gastroenterology, Meizhou People’s Hospital, Meizhou Academy of Medical Sciences, Meizhou, China; 6 Department of Gastroenterology, Shenzhen Qianhai Taikang Hospital, Shenzhen, China

**Keywords:** abatacept, autoimmune enteropathy, CTLA4 haploinsufficiency, primary immunodeficiencies, regulatory T cells

## Abstract

**Background:**

Cytotoxic T-Lymphocyte-Associated Protein 4 (CTLA4) haploinsufficiency is a rare monogenic immunodysregulatory disorder that can affect multiple organ systems, leading to a combination of autoimmunity, immunodeficiency, and lymphoproliferation. It is caused by heterozygous germline mutations of *CTLA4*, yet the structure-function relationships underlying individual pathogenic variants remain incompletely understood.

**Methods:**

We performed whole-exome sequencing on a patient with adolescent-onset autoimmune enteropathy, early-onset gastric malignancy and multisystem comorbidities, who was initially misdiagnosed with Crohn’s disease and refractory to multiple biologics. Functional assays assessed CTLA4 dimerization and CD80/CD86 binding capacity. Peripheral blood immunophenotyping was conducted using CyTOF and flow cytometry. Intestinal histopathology was evaluated by immunohistochemistry. The patient subsequently received targeted therapy with abatacept.

**Results:**

A novel heterozygous missense variant in *CTLA4* (c.167T>G, p.Phe56Cys) was identified. Functional assays demonstrated that this variant impairs CTLA4 protein dimerization and its binding capacity to CD80/CD86, establishing loss-of-function as the molecular basis of pathogenicity. Comprehensive peripheral blood immunophenotyping by CyTOF revealed a broad adaptive immune dysregulation landscape, characterized by depletion of regulatory T cells (Tregs), γδ T cells, and CD161+ cytotoxic-like CD8+ T cells. Flow cytometry confirmed reduction in CD4+CD25+Foxp3+ Treg cells along with decreased CTLA4 expression. Intestinal histopathology showed crypt apoptosis with T cell-predominant infiltration, distinct from classic inflammatory bowel disease. Notably, targeted therapy with abatacept partially restored Treg frequency and alleviated clinical symptoms.

**Conclusion:**

These findings identify p.Phe56Cys as a novel loss-of-function CTLA4 variant that disrupts protein dimerization and ligand binding, expanding the mechanistic understanding of CTLA4 haploinsufficiency and supporting genetic screening in patients with refractory autoimmune enteropathy and early-onset gastric malignancy.

## Introduction

Inborn errors of immunity (IEI) are a heterogeneous group of inherited genetic disorders that impair the immune system, causing increased susceptibility to immunodeficiency, infections, autoimmunity, autoinflammation, and malignancy (1). A large proportion of IEI cases remain unrecognized in their early stages, resulting in substantial diagnostic delays and lost opportunities for timely optimal treatment (2). Notably, more than 20% of IEIs may present with inflammatory bowel disease (IBD)-like intestinal inflammation, and the condition is often refractory to standard IBD treatments, further adding complexity to the management of IEI (3, 4). A distinct subset of IEI is characterized by the simultaneous presence of immune system impairments and other extra-immunological manifestations, for instance, developmental delays, endocrine, nervous or gastrointestinal disorders (5, 6). These conditions usually stem from intricate genetic variants affecting various physiological pathways, resulting in a combined picture of primary immunodeficiency and an extended clinical spectrum. Cytotoxic T-Lymphocyte Antigen 4 (CTLA4) haploinsufficiency, an autosomal dominant IEI first reported by two independent studies in 2014 (7, 8), arises from heterozygous germline variants or deletions in the *CTLA4* gene. As a key negative regulator of T cell activation, CTLA4 maintains immune tolerance primarily through two mechanisms: competitive binding to CD80/CD86 ligands on antigen-presenting cells, and transendocytosis of these ligands, a process predominantly mediated by regulatory T cells (Tregs) (9, 10). CTLA4 haploinsufficiency disrupts this regulatory balance, leading to T cell hyperactivation, Treg dysfunction, and a pleiotropic clinical phenotype characterized by autoimmunity, immunodeficiency, and lymphoproliferation. Importantly, CTLA4 haploinsufficiency exemplifies a primary immunodeficiency with multi-organ involvement, as affected individuals can present with gastrointestinal, dermatologic, neurological and endocrinological abnormalities, as well as increased susceptibility to malignancy (11, 12).

Despite extensive characterization of the clinical manifestations of CTLA4 haploinsufficiency, the precise structure-function relationships underlying individual pathogenic variants remain incompletely understood. The condition exhibits marked clinical heterogeneity with highly variable phenotypes even among intrafamilial cases, which hampers disease identification based solely on clinical symptoms and often results in diagnostic delays (13). Furthermore, the gastrointestinal manifestations of CTLA4 haploinsufficiency have been relatively underrecognized in clinical practice, despite occurring in as high as 62% of patients (13). These manifestations often mimic difficult-to-treat IBD (14), present as early-onset Crohn’s disease (15) or autoimmune enteropathy (16). However, these clinical descriptions do not fully capture the molecular heterogeneity of CTLA4 deficiency, and functional characterization of novel variants is essential for establishing pathogenicity and guiding mechanism-based therapy. The recent introduction of abatacept, a recombinant CTLA4-Ig fusion protein that restores competitive ligand binding, represents a paradigm of mechanism-based treatment for CTLA4 haploinsufficiency (17). Yet, successful application of such targeted therapy requires timely molecular diagnosis and functional validation of the underlying genetic variant.

Herein, we report the identification and functional characterization of a novel heterozygous germline missense variant in *CTLA4* (c.167T>G, p.F56C) in a patient with adolescent-onset autoimmune enteropathy, early-onset gastric malignancy and multiorgan manifestations. Initially misdiagnosed with Crohn’s disease, the patient failed to respond to multiple biologics (including anti-TNF-α agents). We demonstrate that this variant impairs CTLA4 dimerization and subsequently diminishes its avidity for CD80/CD86, leading to loss-of-function. Using comprehensive immune profiling by mass cytometry (CyTOF) and flow cytometry, we define the associated immune dysregulation landscape, characterized by reduced CTLA4 expression in predominantly T cells and decreased frequency of Tregs. Consistent with this systemic immune dysregulation, the patient’s intestinal mucosa showed prominent T cell lymphoproliferation, a pathological hallmark of CTLA4 haploinsufficiency-associated intestinal inflammation. Finally, we show that targeted therapy with abatacept partially improves these immune abnormalities and alleviates clinical symptoms, providing proof-of-concept for mechanism-driven treatment of this novel *CTLA4* variant.

## Results

### Clinical presentation and diagnostic trajectory

**Figure 1:**
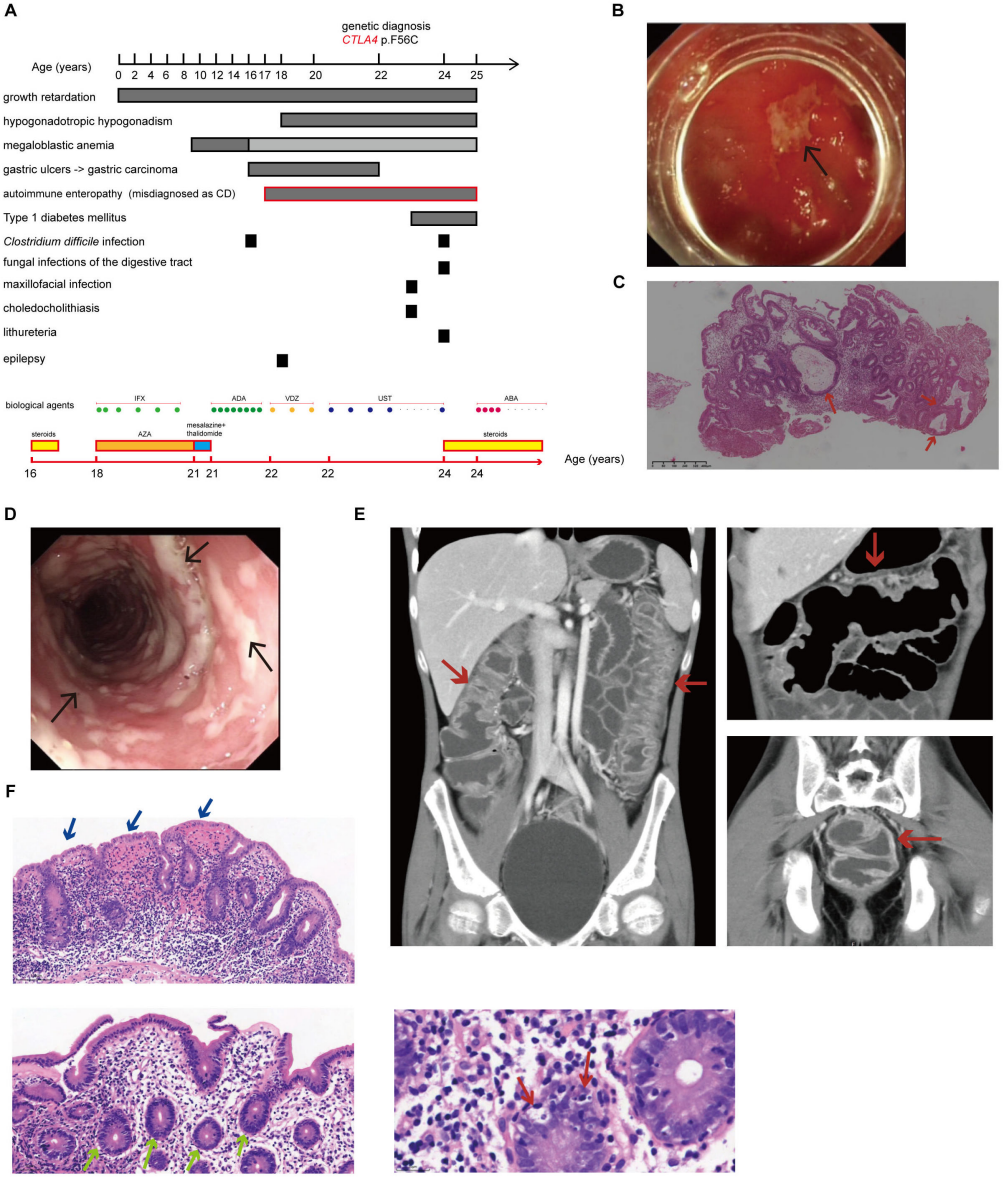
Clinical characteristics of a patient with autoimmune enteropathy harboring a *CTLA4* variant. **(A)** Clinical course of the patient, including the treatment timeline following initial misdiagnosis of Crohn’s disease (CD) IFX: infliximab; ADA: adalimumab; VDZ: vedolizumab; UST: ustekinumab; ABA: abatacept; AZA: azathioprine. **(B)** Gastroscopic image showing ulcers (black arrow) in the gastric body. **(C)** Hematoxylin and eosin (HE)-stained section of the patient’s gastric body showing dilated glands (red arrows). **(D)** Colonoscopic image demonstrating mucosal hyperemia, edema, and purulent secretions (black arrows). **(E)** Computed tomography enterography (CTE) images of the patient, revealing intestinal wall thickening in the ileocecum, ascending colon (left), transverse colon (upper right), and rectum (lower right), marker by red arrows. **(F)** HE-stained colonoscopic biopsy specimens showing chronic active ileitis (upper left, blue arrows indicate villous atrophy), chronic active colitis (lower left, green arrows indicate crypts depleted Paneth cells and goblet cells), and epithelial apoptotic bodies (right, indicated by red arrows).

A 25-year-old male was admitted with persistent watery diarrhea and abdominal pain. At age 9, he presented with growth retardation and skin pallor, diagnosed with complete growth hormone deficiency and autoimmune hemolytic anemia (**Figure 1A**). At age 17, he developed pasty diarrhea and mild periumbilical pain. Gastroscopy revealed multiple gastric body ulcers (**Figure 1B**), characterized by chronic gastritis and glandular destruction by histopathological assessment (**Figure 1C**). Colonoscopy showed mucosal erythema, edema, and purulent discharge involving the terminal ileum and entire colon (**Figure 1D**). Abdominal and pelvic computed tomography (CT) demonstrated significant wall thickening from ileocecum to rectum (**Figure 1E**). Initial testing for common pathogens (*H. pylori* and *C. difficile*) was positive. The patient received a 14-day course of oral vancomycin (250 mg four times daily) for *C. difficile* infection, which cleared the stool toxin positivity, but his diarrhea and abdominal pain did not improve. *H. pylori* eradication therapy likewise failed to alleviate his symptoms.

The patient was suspected to have Crohn’s disease. Despite subsequent aggressive sequential therapy with conventional (mesalamine, azathioprine, thalidomide) and biological agents (infliximab, adalimumab, vedolizumab, and ustekinumab), his symptoms progressed over time (**Figure 1A**). He developed persistent high-output watery diarrhea, severe malnutrition, and profound weight loss, requiring continuous nasogastric enteral nutrition and intermittent parenteral nutrition. He also required multiple hospitalizations due to multiorgan complications, including recurrent *C. difficile* infection, gastrointestinal fungal infection, acute maxillofacial infection, choledocholithiasis, ureteral calculi, epilepsy, and type 1 diabetes mellitus (**Figure 1A**).

The patient experienced three generalized tonic-clonic seizures at age 18 over a limited peroid. Electroencephalography (EEG) showed nonspecific slowing without epileptiform discharges. Brain magnetic resonance imaging (MRI) revealed no significant abnormalities in the brain parenchyma. The epilepsy was classified as idiopathic generalized and well-controlled with low-dose levetiracetam. At age 22, his gastric ulcer underwent malignant transformation, prompting treatment with endoscopic submucosal dissection followed by subtotal gastrectomy, and histologically confirmed as poorly differentiated tubular adenocarcinoma of the stomach. Re-review of intestinal pathology specimens revealed chronic active ileitis and colitis with intestinal villous atrophy, crypt shortening, loss of intestinal Paneth cells and goblet cells, lymphocytic infiltration, and prominent epithelial apoptotic bodies (**Figure 1F**), leading to a revised diagnosis of autoimmune enteropathy (18) rather than Crohn’s disease. Basic immunological workup revealed decreased serum immunoglobulin levels (including IgG and IgA) (**Supplementary Table 1**). Lymphocyte subset analysis showed reduced percentage and absolute number of CD19+CD45+ B cells compared to reference range (**Supplementary Table 2**), indicating an impaired humoral immune response. Serum cytokine profiling revealed elevated levels of pro-inflammatory cytokine Interleukin-6 and the immunoregulatory cytokine IL−10, while IL−2, IL−4, Tumor necrosis factor-α, interferon‑γ, and IL−17A were within normal ranges (**Supplementary Table 3**).

### Identification of a novel inherited *CTLA4* variant

**Figure 2:**
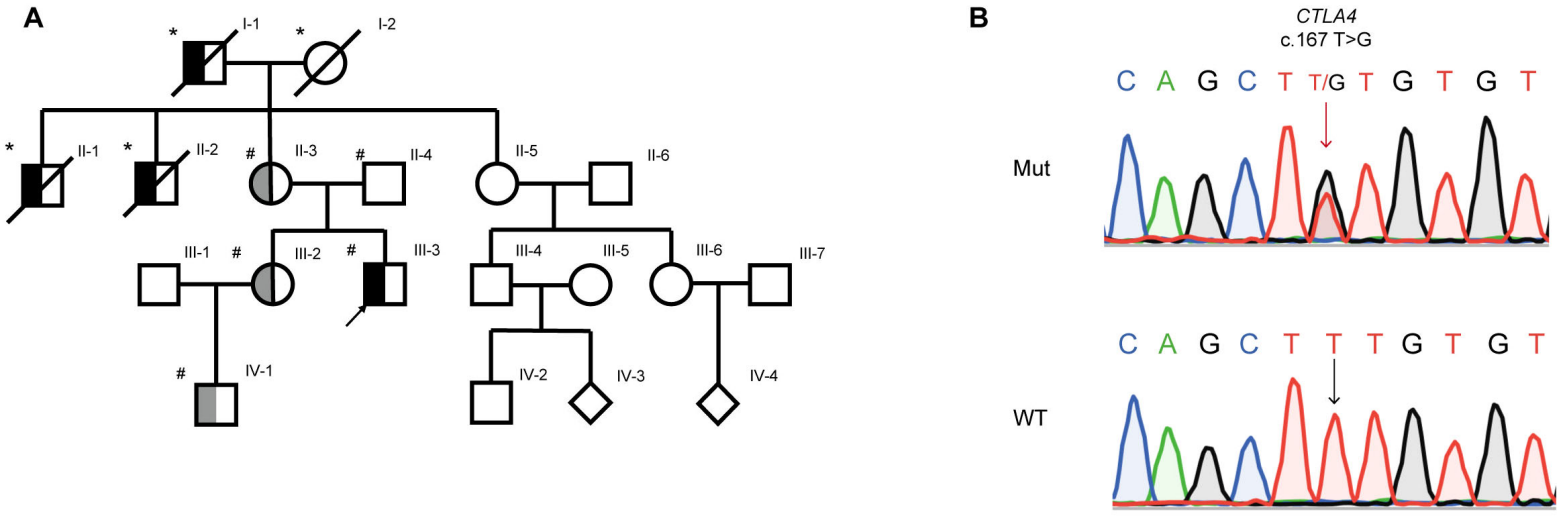
Identification of a novel inherited *CTLA4* variant. **(A)** Pedigree of the patient’s family. Squares represent male subjects; circles represent female subjects; diamonds represent fetuses of unknown gender. Black half-filled symbols indicate patients with heterozygous variants; grey half-filled symbols indicate asymptomatic carriers with heterozygous variants; crossed-out symbols indicate deceased subjects; asterisks indicate genotypes inferred based on clinical manifestations; pound signs indicate variants validated by genomic DNA sequencing; arrows indicate the proband. Genotyping of II-5 and her offspring for this variant requires further validation. **(B)** Sanger sequencing validation of the novel heterozygous *CTLA4* variant.

The coexistence of refractory diarrhea, autoimmune enteropathy, early-onset gastric malignancy, recurrent infections, hypogammaglobulinemia and multisystem comorbidities in this young adult constitutes a constellation of clinical features highly suggestive of a monogenic form of primary immunodeficiency (19), which prompted us to initiate pedigree screening and targeted Sanger testing (**Figure 2A**). The index patient (III-3) was born to a non-consanguineous family. His maternal grandfather (I-1) died at age 69 due to bile duct obstruction. His mother (II-3) and sister (III-2) were asymptomatic, while two maternal uncles (II-1 and II-2) had died in adolescence of unknown or suspected intestinal malignancy. Whole-exome sequencing of the patient’s peripheral blood identified a previously unreported heterozygous missense variant in exon 2 of the *CTLA4* gene, NM_005214.5(CTLA4): c.167T>G (p.Phe56Cys). Per the American College of Medical Genetics and Genomics (ACMG) guidelines (20), this variant was initially classified as a variant of uncertain significance (VUS). Subsequent Sanger sequencing validation across all available family members confirmed the presence of this *CTLA4* variant and demonstrated its inheritance from his mother (II-3) (**Figure 2B**). The patient’s sister (III-2) and 6-year-old nephew (IV-1) also carry this variant in a heterozygous manner, yet both remain asymptomatic to date, indicating incomplete penetrance of the newly identified *CTLA4* variant.

### *CTLA4* Phe56Cys variant impairs protein dimerization and ligand binding

**Figure 3:**
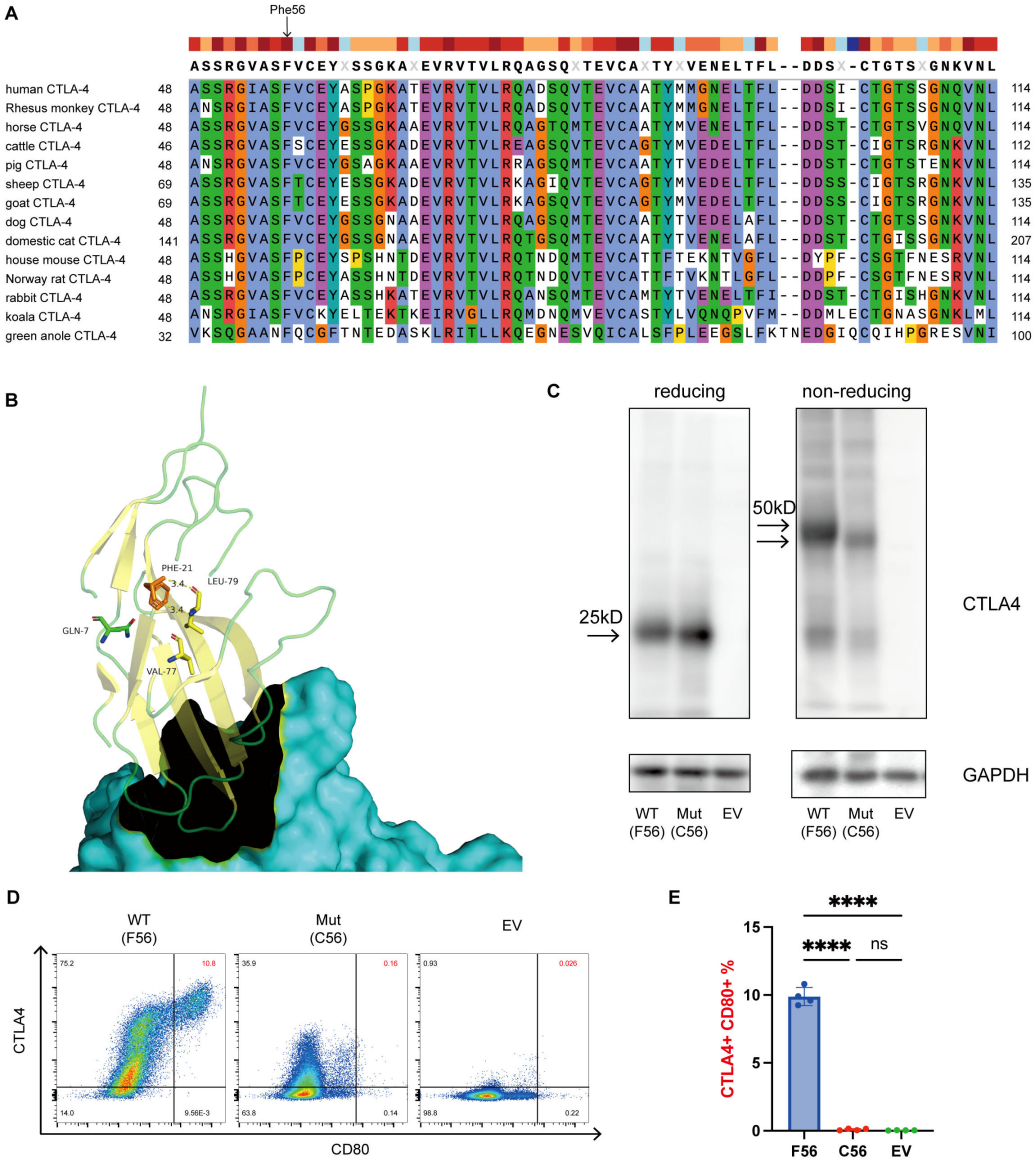
The *CTLA4* p.Phe56Cys (F56C) variant impairs CTLA4 dimerization and ligand-binding capability. **(A)** Multiple-sequence alignment of the CTLA4 protein (residues 48–114) from humans and other chordate species. **(B)** Structural model of the CTLA4/CD80 complex. CTLA4 is shown in a cartoon model, and CD80 is shown in a teal surface model. The Phe56 residue (orange stick model, denoted as “PHE-21” in the image) forms tight packing with Gln42, Val112, and Leu114 (4Å around Phe60, GLN-7, PHE-21 and LEU-79 in image, respectively). Additionally, Phe56 forms hydrogen bonds with Leu114 to stabilize the CTLA4 structure. **(C)** Immunoblot analysis of CTLA4 and GAPDH (loading control) in HEK293T cells transfected with plasmids encoding wild-type CTLA4 (F56), the CTLA4 p.Phe56Cys variant (C56), or an empty vector (EV), under both reducing and non-reducing conditions. **(D)** Flow cytometric analysis of CTLA4 expression and CD80-binding capability in HEK293T cells transfected with wild-type CTLA4 (F56), the CTLA4 p.Phe56Cys variant (C56), or empty vector (EV). **(E)** Quantification of CD80-binding proportions, shown as percentage of CTLA4+CD80+ cells among all live HEK293T cells. ****, P<0.0001.

We next sought to identify the functional impact of the *CTLA4* p.Phe56Cys variant. Multiple-sequence alignment of CTLA4 protein sequences across vertebrate species revealed that the CTLA4 Phe56 is an evolutionarily highly conserved, non-polar and hydrophobic amino acid in the extracellular ligand-binding domain of CTLA4 (**Figure 3A**). The Combined Annotation-Dependent Depletion (CADD) phred score for this variant is 25.1, which is above the deleterious threshold of 20, supporting its likely pathogenic effect in *silico (*21). Structural modeling predicted that the Phe56 residue is tightly packed with Gln42, Val112 and Leu114. Together, these residues contribute to the formation of a β-sheet, a key structural element that supports the assembly of CTLA4’s immunoglobulin-like extracellular domain, and important for maintaining CTLA4’s structural integrity and functional competence. The variant Phe56Cys replaces the hydrophobic phenylalanine with a more polar cysteine and is predicted to disrupt local hydrophobic packing, destabilize the β-sheet and overall CTLA4 structure, and potentially impair its ability to interact with its cognate ligand CD80 (**Figure 3B**). The covalent dimerization of CTLA4 is essential for its high-avidity binding to the costimulatory ligands CD80 and CD86 on antigen-presenting cells, which in turn enables CTLA4 to regulate T cell activation and ultimately contribute to the maintenance of immune tolerance (22). To directly assess whether the p.Phe56Cys variant impairs the intrinsic ligand-binding capacity of CTLA4, we performed a binding assay using soluble CD80-Fc and CD86-Fc fusion proteins in HEK293T cells. Consistent with our structural predictions, ectopic expression of the CTLA4 p.Phe56Cys variant rather than the wild-type CTLA4 in HEK293T cells resulted in reduced CTLA4 dimerization under non-reducing sodium dodecyl sulfate-polyacrylamide gel electrophoresis (SDS-PAGE) conditions (which allows visualization of disulfide bond-dependent dimers, see Methods), without significant change in total CTLA4 protein levels (under reducing condition) (**Figure 3C**). Notably, flow cytometric analysis showed that transfection of the CTLA4 p.Phe56Cys plasmid led to a significant decrease in CTLA4’s binding to CD80 (**Figure 3D**), shown as a substantial reduction in the percentage of CTLA4+CD80+ cells among all live HEK293T cells (**Figure 3E**). The same phenomenon was also observed for the other cognate ligand CD86 (**Supplementary Figures 1A, B**). Interestingly, we also observed a significant reduction in the surface expression of CTLA4 on HEK293T cells (**Figure 3D**). Current literature indicates that CTLA4 is primarily an intracellular antigen whose surface expression is tightly regulated by restricted trafficking to the cell surface and rapid internalization (23, 24). We hypothesize that the misfolded protein caused by the Phe56Cys variant may be retained inside the cell or prone to degradation, leading to a marked decrease in CTLA4 protein transported to the cell surface, which merits further investigation. Collectively, these data demonstrate that the p.Phe56Cys variant causes CTLA4 loss-of-function by impairing dimerization and reducing cell surface expression, which in turn diminishes its capacity to bind CD80/CD86.

### Abnormal immune landscape associated with *CTLA4* Phe56Cys variant

**Figure 4:**
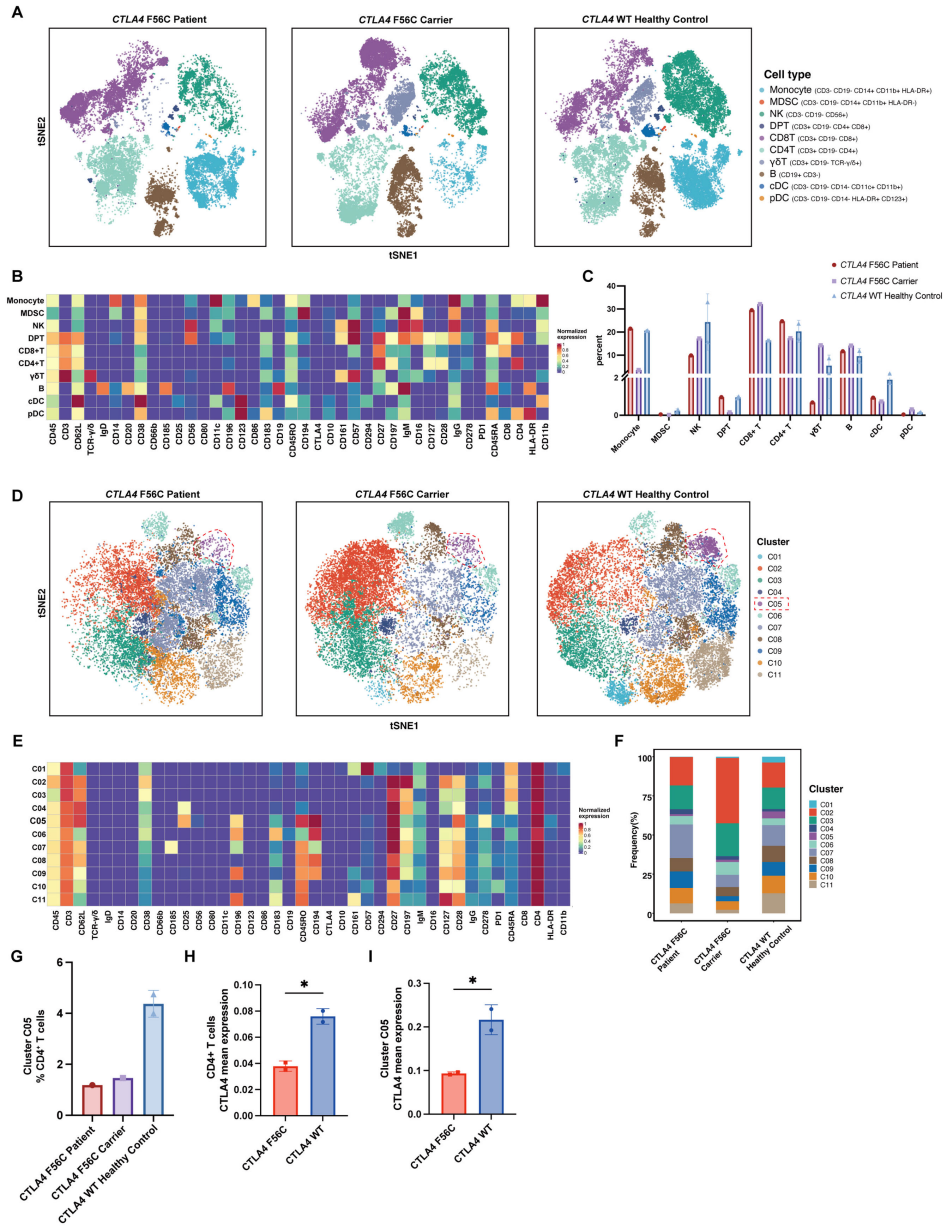
The *CTLA4* p.Phe56Cys variant is associated with an aberrant peripheral immune landscape. **(A)** t-distributed stochastic neighbor embedding (tSNE) plots of peripheral blood mononuclear cells (PBMCs) from three groups: the CTLA4 p.Phe56Cys variant patient, his nephew (an asymptomatic carrier of the same variant), and two healthy controls. Cells are colored according to cell type. **(B)** Heatmap showing marker expression profiles of the cell types identified in **(A)**. Each row represents the median arcsine-transformed intensities (each marker was scaled by percentiles) in each cell type. **(C)** Quantification of cell type proportions among PBMCs from the three groups (corresponding to **(A)**). **(D)** tSNE plots of CD4+ T cells from the CTLA4 p.Phe56Cys patient, asymptomatic nephew, and healthy controls, showing distinct clustering patterns. **(E)** Heatmap showing marker expression profiles of the CD4+ T cell clusters identified in **(D)**. **(F)** Quantification of CD4+ T cell cluster proportions in the three groups (corresponding to **(D)**). **(G)** Percentage of Cluster C05 (CD25+CD194+CD127- regulatory T cells) among CD4+ T cells in the three groups. **(H, I)** CTLA4 expression levels (quantified by median arcsine-transformed intensities) in total CD4+ T cells **(H)** and CD25+CD194+CD127- Tregs **(I)** from CTLA4 p.Phe56Cys carriers (patient and asymptomatic nephew, n=2) and CTLA4-wild-type healthy controls (n=2). *, *P*<0.05.

Given the role of CTLA4 in regulating immune responses, particularly T cell function (9), we aimed to comprehensively characterize the immune consequences of the *CTLA4* p.Phe56Cys variant. We performed high-dimensional cytometry by time of flight (CyTOF) analysis on PBMCs derived from the index patient, his asymptomatic carrier nephew (IV-1), and two age- and gender- matched healthy controls. A 40-marker CyTOF panel was used for detection (**Supplementary Table 3**). Via X-shift clustering (25), cells were categorized into 10 major distinct clusters (**Figure 4A**) and 26 subclusters (**Supplementary Figure 2A**) based on their relevant marker expression profiles (**Figure 4B**; **Supplementary Figure 2B**). Among innate immune subsets, the frequencies of monocytes and myeloid-derived suppressor cells (MDSCs) were comparable between the patient and healthy controls. In contrast, the proportion of natural killer (NK) cells and conventional dendritic cells (cDC) were relatively lower in the patient (**Figure 4C**). Among the adaptive immune cell subsets, no significant differences in the frequencies of B cells or CD4+CD8+ double-positive T cells were observed between the patient and healthy controls. Notably, γδ T cells, a subset critical for rapid pathogen defense and tissue homeostasis, were remarkably reduced in the patient, but not in the asymptomatic carrier, when compared to the healthy controls (**Figure 4C**). Within CD8+ T cells, the patient showed expansion of naïve populations (defined as CD45RA+CD197+CD27+CD161-CD62L+CD127+ cells, cluster C12, **Supplementary Figure 2C**) and reduction of cytotoxic-like CD8+ T cells (CD161+CD127+, cluster C05); notably, the CD161+ cytotoxic-like CD8+ T cell reduction was also present in the asymptomatic carrier (**Supplementary Figures 2C–E**). CD4+ T cells were further divided into 11 subsets (**Figures 4D, E**). Compared to the healthy controls, the patient’s CD4+ T cells showed evidence of incomplete differentiation, as the frequency of CD57+CD4+ T cells (cluster C01, **Figure 4E**), a marker of terminal differentiation and effector function (26) was reduced. Furthermore, two functionally relevant CD4+ T subsets were decreased. One is the CD127+CD4+ T cells (cluster C11), critical for long-term pathogen-specific immunity and inversely correlates with FoxP3 and suppressive function of human CD4+ Treg cells (27). The other is CD196+CD4+ T cells (cluster C09 and C11), which express the chemokine receptor CCR6 and mediate T cell migration to inflammatory sites (28) (**Figures 4E, F**). Notably, CD4+ T cell analysis revealed a profound reduction in Tregs. Both the patient and the asymptomatic carrier exhibited an approximately 4-fold reduction in the CD25+CD194+CD127- Treg subset (**Figure 4G**, circled as “C05” in **Figure 4D**), a population conveying effective suppressive function in human peripheral blood (29). Since CTLA4 is a constitutively expressed negative regulator in Tregs that mediates inhibitory function (30), we examined CTLA4 expression across cell subsets (**Supplementary Figures 2F, G**). Consistent with the Treg reduction, CTLA4 expression, measured by median arcsinh-transformed metal intensity, was significantly decreased in the patient’s total CD4+ T cells (**Figure 4H**) and more prominently in his Tregs (**Figure 4I**). The asymptomatic nephew showed a similar reduction in CTLA4 expression. Collectively, these data demonstrate that the *CTLA4* p.Phe56Cys variant is associated with aberrant adaptive immune profiles, with a core immune signature of Treg depletion and reduced CTLA4 expression.

### *CTLA4* Phe56Cys variant led to abnormal Treg cell phenotype and intestinal lymphocytic infiltration

**Figure 5:**
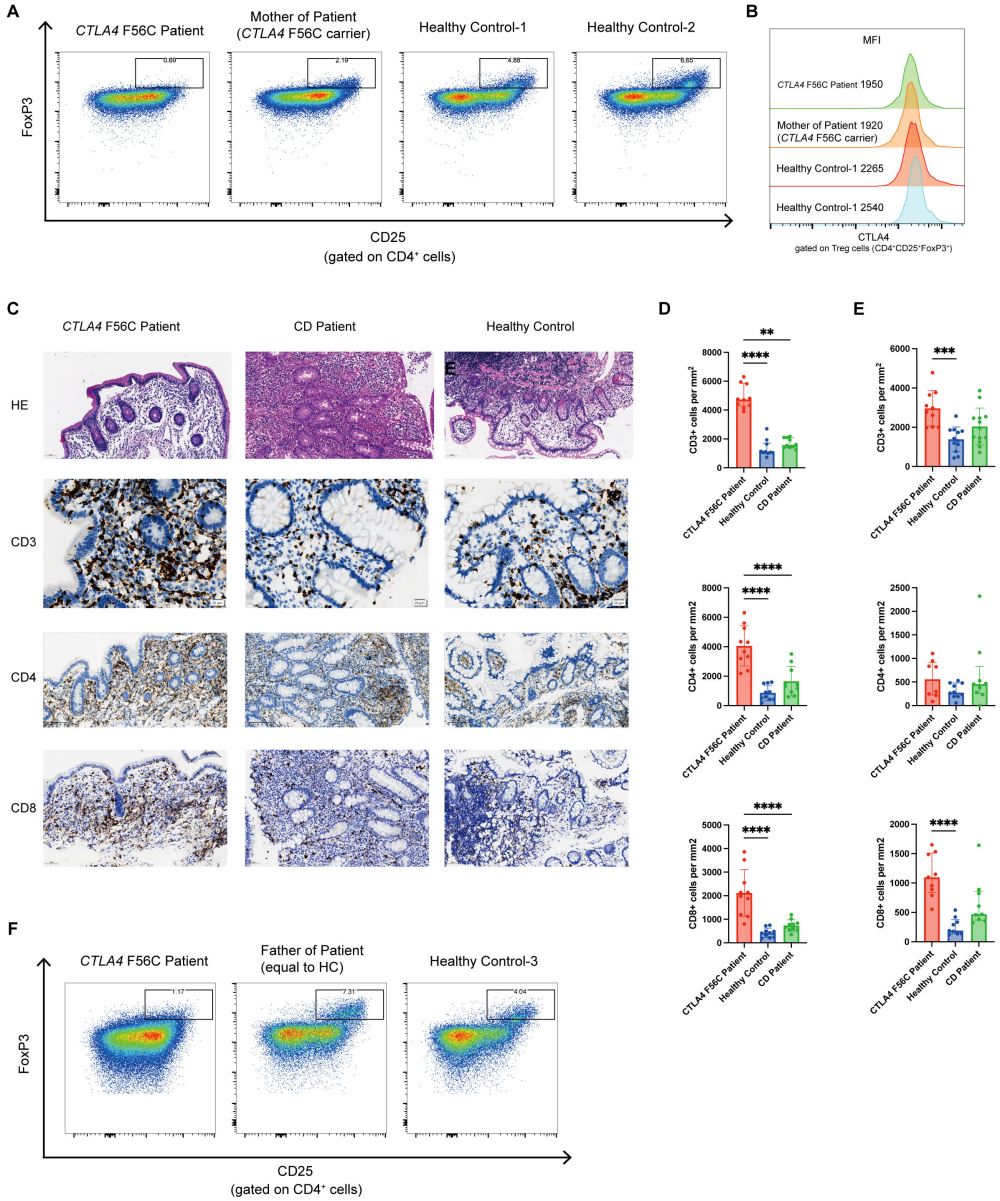
The *CTLA4* p.Phe56Cys (F56C) variant contributes to abnormal Treg phenotype and intestinal lymphocytic infiltration. **(A)** Flow cytometric analysis of FoxP3+CD25+ Treg cells within the CD4+ T cell population from PBMCs of the CTLA4 p.Phe56Cys variant patient, mother of the patient (asymptomatic carrier) and healthy controls. **(B)** CTLA4 expression in Treg cells (gated as CD4+FoxP3+CD25+ cells) from the three groups in **(A)**, and quantified median fluorescence intensity (MFI) of CTLA4 expression is marked. **(C)** Histopathological staining of terminal ileum biopsy specimens from the CTLA4 p.Phe56Cys patient, a Crohn’s disease (CD) patient, and a healthy control. Staining types include hematoxylin and eosin (HE) for general tissue morphology, CD3 immunostaining for total T cells, CD4 and CD8 immunostaining for CD4+ and CD8+ T cells, respectively. **(D, E)** Quantitative comparison of CD3+ cell (upper), CD4+ cell (middle) and CD8+ cell (lower) infiltration in the submucosa **(D)** and intraepithelial compartment **(E)** of terminal ileum tissues from the three groups in **(C)**. Each dot represents a single counting area. **(F)** Flow cytometric analysis of CD4+FoxP3+CD25+ Treg cells in PBMCs from the CTLA4 p.Phe56Cys patient (after 6 months of abatacept treatment) and CTLA4-wild-type healthy controls (n=2). **, P<0.01; ***, P<0.001; ****, P<0.0001.

Prior studies have shown that global CTLA4 deletion in mice impairs the suppressive function of Treg cells, leading to severe autoimmune disease and early lethality (31). To gain more insight into the impact of *CTLA4* Phe56Cys variant on human Treg cells, we performed flow cytometric analysis of peripheral blood Tregs. Consistent with our CyTOF findings, the patient exhibited an evident reduction in the percentage of CD4+CD25+Foxp3+ Treg cells in the peripheral blood compared to age- and gender-matched healthy controls (**Figure 5A**). The patient’s mother (II-3), an asymptomatic carrier of the same variant, had a moderately higher percentage of Treg cells than the patient, though still lower than that of healthy controls. Furthermore, the expression level of CTLA4 in Treg cells (quantified by median fluorescence intensity) was decreased in both the patient and his mother, relative to controls (**Figure 5B**). Treg cells are critical for maintaining immune tolerance and intestinal mucosal homeostasis (32, 33). Given the patient’s prominent intestinal manifestations including refractory diarrhea and autoimmune enteropathy, we performed immunohistochemistry (IHC) on terminal ileum tissue to assess whether Treg deficiency correlates with local intestinal lymphocytic infiltration. Hematoxylin-eosin (HE) staining showed prominent inflammatory cell infiltration in the terminal ileum of both the *CTLA4* p.Phe56Cys patient, compared to a non-inflammatory healthy control and a Crohn’s disease (CD) patient who had active disease at the time of biopsy (with approximately 2 months of disease duration) and had not received biologic therapy (**Figure 5C**). Notably, IHC analysis revealed that the CTLA4 mutated patient had markedly increased infiltration of T cell markers (CD3+, CD4+ and CD8+ cells) in the intestinal submucosa, exceeding that observed in both the CD patient and healthy controls (**Figure 5D**). Intraepithelial lymphocytes (IELs) also exhibited increased CD3+ and CD8+ cell infiltration in the patient, whereas CD4+ intraepithelial lymphocytes remained comparable to healthy control (**Figure 5E**). Collectively, these results suggest a dysregulated intestinal T cell response associated with the *CTLA4* p.Phe56Cys variant, characterized by systemic Treg deficiency and localized intestinal T cell infiltration.

### Targeted abatacept treatment leads to partial clinical and biological response

Having determined CTLA4 haploinsufficiency (caused by the novel p.Phe56Cys variant) as the underlying cause of the patient’s symptoms, and given the failure of prior biologic therapies, we initiated targeted therapy with abatacept, a recombinant fusion protein that mimics CTLA4’s immunomodulatory function. It consists of the CTLA4 extracellular domain fused to the Fc fragment of human IgG1, enabling it to compete with endogenous dysfunctional CTLA4 for binding to CD80/CD86 (34). The patient received abatacept administered subcutaneously at 10 mg/kg every 2 weeks for 6 months, combined with low-dose oral methylprednisolone (8 mg daily). Clinically, he reported partial resolution of gastrointestinal symptoms, in which diarrhea frequency and volume decreased, and weight gain was observed. Correspondingly, his Harvey-Bradshaw Index, a validated surrogate for assessing colitis disease activity (35), dropped from 24 to 10, indicating moderate improvement in disease severity. To evaluate whether abatacept restored the underlying immune abnormalities, we performed post-treatment flow cytometry. After 6 months of abatacept treatment, the frequency of CD4+CD25+Foxp3+ Treg cells in his peripheral blood increased compared to pretreatment levels, though it remained below healthy control values (**Figure 5F**). This partial restoration of Treg frequency correlated with the observed clinical response. Collectively, these data demonstrate that targeting the CTLA4 pathway with abatacept led to partial clinical and biological improvement in the patient, providing proof-of-concept for mechanism-based therapy.

## Discussion

In this study, we identify a novel *CTLA4* missense variant (c.167T>G, p.Phe56Cys) that causes loss-of-function by impairing protein dimerization and CD80/CD86 binding. This molecular abnormality leads to a core immune signature of Treg depletion and systemic adaptive immune dysregulation, which may be associated with the multi-system manifestations observed in this patient, including autoimmune enteropathy, early-onset gastric malignancy, hypogammaglobulinemia, growth retardation, and possibly endocrinopathies. Whether the patient’s epilepsy was directly related to the *CTLA4* variant remains unclear, as brain MRI showed no evidence of the inflammatory or demyelinating lesions typically associated with CTLA4 haploinsufficiency (36), and the patient had concurrent pulmonary infection and was receiving cefoperazone-sulbactam, a β-lactam antibiotic known to lower seizure threshold, making it difficult to definitively attribute the seizures to the *CTLA4* variant.

Gastrointestinal involvement occurs in up to 61% of CTLA4 haploinsufficiency patients, and key clinical hallmarks of CTLA4-related enteropathy often include chronic diarrhea, malabsorption, failure to thrive and protein-losing enteropathy (13). Previous reports have noted that gastrointestinal manifestations of CTLA4 haploinsufficiency are often severe and clinically mimic difficult-to-treat IBD (14), underscoring the importance of genetic testing in patients with refractory intestinal inflammation to distinguish monogenic etiologies from idiopathic IBD (37). Our patient exemplifies this diagnostic challenge. It was through *CTLA4* genetic sequencing and supportive immune phenotyping that the correct diagnosis of this monogenic immunodysregulation disorder was confirmed. Critically, in our patient, CTLA4 haploinsufficiency is characterized by a T cell-mediated autoimmune enteropathy, exhibiting distinct pathological features that include crypt apoptosis and a mononuclear cell infiltrate composed primarily of T lymphocytes but not neutrophils. This profile closely resembles the pathology of graft-versus-host disease (GVHD) (38) or immune checkpoint inhibitor (ICPI)-induced colitis (39), rather than classic IBD. This unique apoptotic pattern directly stems from uncontrolled cytotoxic T cell activation against intestinal epithelial cells, a consequence of impaired CTLA4-mediated immune inhibition (33, 40). Thus, CTLA4 haploinsufficiency should be considered in any patient with an “autoimmune type” enteropathy refractory to conventional IBD therapies, especially when it co-occurs with extraintestinal features of immune dysregulation like hypogammaglobulinemia, autoimmune cytopenia, or lymphoproliferation.

Notably, CTLA4 haploinsufficiency is amenable to mechanism-targeted therapy. Abatacept, a recombinant CTLA4-Ig fusion protein, restores the “missing brake” on T cell activation, which has substantially improved outcomes in affected patients (41, 42). Our patient achieved a partial clinical and immunological response to abatacept, with a moderate reduction in disease activity and a partial increase in peripheral Treg frequency. Importantly, we interpret the observed Treg partial recovery as a secondary consequence of reduced T-cell activation following CD28 blockade, rather than a direct Treg-specific effect of abatacept. We acknowledge that the absence of follow-up colonoscopy and fecal calprotectin data after abatacept treatment has limited a more comprehensive assessment of intestinal mucosal healing. The mechanisms underlying non-responsiveness or incomplete responses to abatacept remain poorly characterized. Suspected contributing factors include unestablished optimal dose, timing, or duration of abatacept treatment (17, 42), as well as the activation of alternative immune pathways independent of the CTLA4/B7 axis. For patients with suboptimal responses to abatacept, future therapeutic considerations should include hematopoietic stem cell transplantation (43) or additional immunosuppressants (e.g., sirolimus) that may address more severe or refractory CTLA4-mediated immune dysregulation and enteropathy (17).

Incomplete penetrance is a hallmark of CTLA4 haploinsufficiency, with an estimated penetrance of approximately 67% (13). The patient’s mother, sister and nephew carry the p.Phe56Cys variant but remain asymptomatic; both the mother and the nephew exhibit reduced CTLA4 expression and Treg alterations, despite being clinically asymptomatic. We acknowledge that different asymptomatic carriers were used for CyTOF and flow cytometry analyses due to sample availability constraints. However, the convergent immune alterations observed in these two independent carriers strengthen the validity of our findings. These observations suggest the existence of genetic modifiers that modulate the severity of CTLA4-mediated immune dysregulation, thereby influencing clinical penetrance. Indeed, one study has identified a patient with CTLA4 haploinsufficiency who carried a maternally inherited pathogenic *CTLA4* variant and another paternally inherited rare loss-of-function missense variant in *CLEC7A*. The *CLEC7A* variant caused partial deficiency of DECTIN-1, a β-glucan pattern recognition receptor, which exacerbated the Treg defect induced by CTLA4 haploinsufficiency, establishing *CLEC7A* as a modifier gene (44). Another study found potential protective or pathogenic single nucleotide variants (SNVs) in unaffected and affected family members of CTLA4 haploinsufficiency respectively. The unaffected carriers harbored a protective SNV in *IL10*, while affected individuals carried an SNV located in *FURIN* gene, both were implicated as genetic modifiers (15). The Human Leukocyte Antigen (HLA) system is known to interact with CTLA4, and potential protective association between HLA-DRB1*01:01 and reduced gastrointestinal symptoms in CTLA4 haploinsufficiency has been reported (45). Beyond the genetic codes, environmental triggers such as infections, intestinal microbiota composition and other exposures, might contribute to the development of the disease in susceptible individuals, warranting further investigations (46, 47). In this family, the observation that male carriers (maternal uncles) died young while female carriers (mother, sister) remain asymptomatic indicates a gender-dependent association with incomplete penetrance. This aligns with literature showing gender-specific associations of *CTLA4* polymorphisms with clinical outcomes, such as in hepatitis C virus (HCV) infection (48). These differences may stem from gender-specific T cell immune regulation, potentially driven by hormonal environments that alter CTLA4 activity or immune response dynamics. Further research is needed to clarify the extent of gender’s influence on disease onset and severity in CTLA4-related disorders. Furthermore, long-term follow-up of asymptomatic carriers, particularly the young nephew, will be essential to clarify the natural history of this variant.

The systemic immune landscape of CTLA4 haploinsufficiency remains incompletely characterized. Through deep and comprehensive immunophenotyping, we identified that the *CTLA4* p.Phe56Cys variant primarily associates with adaptive immune dysregulation, with key abnormalities in multiple T cell subsets, each linked to the patient’s clinical manifestations. The marked depletion of γδ T cells, critical for rapid pathogen recognition, tissue homeostasis maintenance and early anti-infective responses (49), is consistent with the patient’s recurrent infections, a core clinical symptom. The decreased frequency of CD57+, CD127+ and CD196+ CD4+ T cells in the patient collectively suggest a weakened long-lasting immune response to pathogens and reduced T cell chemotaxis function associated with the *CTLA4* variant. More importantly, the combined loss of Treg cells and CTLA4 expression in the patient eliminated a critical immune “brake”, leading to uncontrolled lymphocyte infiltration in multiple organs, most notably the gastrointestinal tract, manifesting as autoimmune enteropathy. Interestingly, the reduction in Treg frequency and CTLA4 expression was also observed in asymptomatic carriers, suggesting that Treg depletion alone is unlikely to be the sole pathogenic driver of clinical disease. Rather, this immune alteration may represent a biomarker or immune correlate of CTLA4 haploinsufficiency. While our finding of reduced Treg frequency in this CTLA4-insufficient patient is consistent with some reports (41, 50), we acknowledge that it differs from observations in large cohort studies, which have commonly reported normal or even elevated Treg frequencies in CTLA4 haploinsufficiency, likely as a compensatory response to chronic immune activation (13, 51). This apparent discrepancy may be explained by the substantial clinical and immunological heterogeneity of CTLA4 haploinsufficiency, which is influenced by genetic modifiers (44), disease stage, and patient-specific heterogeneity, as different CTLA4 mutations may have differential effects on Treg homeostasis (52). Furthermore, functional defects in Treg cells, including impaired CTLA4 expression and suppressive capacity, may be present even when Treg numbers are preserved (7, 11). Nonetheless, this Treg deficiency also represents a putative therapeutic target, as abatacept treatment partially restored the patient’s Treg cell frequency (**Figure 5F**), supporting the potential of monitoring Treg subsets as a biomarker for treatment response. CD161+ cytotoxic-like CD8+ T cells, a subset critical for anti-tumor immunity (53), were reduced in the patient, providing a mechanistic link to the increased malignancy susceptibility of CTLA4 haploinsufficiency. Our patient developed early-onset gastric malignancy, and together with previous reports pointing out elevated rates of gastric cancer and lymphoma in CTLA4 haploinsufficiency (42), these collectively indicates CTLA4’s role in suppressing malignant transformation. We acknowledge that functional studies using patient-derived primary T cells were not performed. Such experiments - including ligand uptake assays, and mixed lymphocyte reactions - would provide more physiologically relevant evidence of the variant’s impact on T-cell function. We recognize this as a limitation and suggest that future studies in newly diagnosed, untreated patients incorporate these standardized approaches to further validate our findings.

In summary, through clinical phenotyping, genetic sequencing, functional validation, and in-depth immunophenotyping, we have identified a novel *CTLA4* loss-of-function variant (p.Phe56Cys) as the etiological cause of autoimmune enteropathy and early-onset gastric malignancy. This variant impairs CTLA4 protein dimerization and ligand binding, leading to a characteristic immune dysregulation signature of Treg depletion and T cell subset alterations. These findings expand the mechanistic understanding of CTLA4 haploinsufficiency and support the consideration of *CTLA4* genetic screening in patients with refractory autoimmune enteropathy, particularly when accompanied by systemic immune dysregulation or early-onset malignancy. From a clinical perspective, the diagnostic delay observed in our patient - spanning over a decade - highlights several actionable strategies to facilitate earlier recognition. First, a lower threshold for genetic testing should be adopted in patients with refractory autoimmune enteropathy who present with extraintestinal features. Second, clinicians should maintain a high index of suspicion for monogenic etiologies in patients who fail to respond to conventional immunosuppressive or biologic therapies. Third, once a clinical suspicion is raised, expedited genetic testing should be pursued to enable timely initiation of mechanism-based therapy. Finally, given the incomplete penetrance observed in CTLA4 haploinsufficiency, cascade screening of asymptomatic family members should be offered, with genetic counseling and longitudinal clinical follow-up for carriers. A multidisciplinary approach involving immunologists, gastroenterologists, and clinical geneticists is essential to optimize early diagnosis and comprehensive management.

## Methods

### Patient and ethic approval

This study was approved by the Human Ethics Committee of the First Affiliated Hospital of Sun Yat-sen University ([2023]514). Informed consent for study procedures and use of their samples for research purpose was obtained from all individual participants included in the study.

### Whole exome sequencing and Sanger sequencing

Genomic DNA was extracted from EDTA-preserved peripheral blood using QIAsymphony DNA Mini Kit (Qiagen, 931236) by QIAsymphony SP (Qiagen). DNA library was constructed using QIAseq FX DNA Library Kit (Qiagen, 180482) and purified using AMPure XP beads (Beckman Coulter). Capture and enrichment of exons were performed using xGen Exome Research Panel (IDT, 1056115). Sequencing of 2 × 150 bp paired-end reads was performed on Novaseq 6000 platform (Illumina). The reads were mapped against human reference genome UCSC hg19 by BWA algorithm, then variants were annotated by PriVar (54) and associated with multiple databases, including 1000genome, ExAC, ESP6500, dbSNP, ClinVar, OMIM, HGMD, and predicted by PolyPhen2, LRT, Mutation Taster, et al. Candidate variant was confirmed by Sanger sequencing on genomic DNA.

### Flow cytometry of PBMC

PBMCs isolation from EDTA-preserved peripheral blood were performed using Ficoll (Cytiva, 17544203) according to the manufacturer’s instruction. Cells were surface stained for 30min at 4°C with following antibodies: CD4 AF647 (Biolegend, clone RPA-T4), CD25 PE (Biolegend, clone BC96). Cells were then washed, fixed and permeabilized with eBioscience Foxp3/Transcription Factor Staining Buffer Set (Invitrogen, 00-5523-00) according to manufacturer’s instruction and stained intracellularly using following antibodies: FOXP3 AF488 (Biolegend, clone 206D), CTLA4 PE-Cy7 (Invitrogen, Clone 14D3). Samples were acquired on Attune NxT Acoustic Focusing Cytometer (Thermo Fisher Scientific) and analyzed using FlowJo Software (v10.8.1, TreeStar).

### Cell lines, plasmids and transfection

HEK293T cells were cultured in DMEM, high glucose (Gibco) with 10% fetal bovine serum (FBS). Wildtype (WT) and point mutation c.167T>G CTLA4 was cloned into pcDNA3.1 by OBiO Tech. The plasmids were transfected into HEK293T cells using Lipofectamine 3000 (Invitrogen) according to the manufacturer’s instruction.

### Immunoblot

Cultured cells were lysed on ice in RIPA lysis buffer (Merck) with protease inhibitor cocktail added (Thermo Scientific). Lysates were separated by sodium dodecyl sulfate-polyacrylamide gel electrophoresis (SDS-PAGE) and transferred onto poly (vinylidene difluoride) membranes. For non-reducing SDS-PAGE, samples were prepared in Laemmli sample buffer (Bio-rad, 1610747) without dithiothreitol (DTT), allowing visualization of disulfide bond-dependent dimers. For reducing condition, Protein SDS PAGE Loading Buffer (Takara, 9173) with dithiothreitol included was used. Immunoblots were performed using the following antibodies: Goat-anti-CTLA4 (R&D, AF-386-PB), Rabbit-anti-GAPDH (Cell Signaling Technology, 2118), Anti-goat IgG, HRP-linked (R&D, HAF017), Anti-rabbit IgG, HRP-linked (Cell Signaling Technology, 7074).

### Immunohistochemistry

Three-μm sections were cut from formalin-fixed, paraffin-embedded samples. Biopsy samples were routinely stained with hematoxylin and eosin. Immunohistochemistry was performed using a horseradish peroxidase-catalyzed brown chromogen reaction in an automated staining system (Leica). Antigen retrieval was performed with EDTA. Antibodies to the following proteins were used: CD3 (MXBiotechnologies, clone MX036), CD4 (MXBiotechnologies, clone MXR036). Images were captured using Digital Pathology Slide Scanner (KFBIO) and quantified using QuPath software (55).

### CD80 and CD86 binding assay

CTLA4 plasmids (WT or c.167T>G) transfected HEK293T cells were detached using Accutase (Invitrogen) and then incubated with HIS-tagged CD80-Fc fusion protein (R&D, 740-B1) and HIS-tagged CD86-Fc fusion protein (R&D, 141-B2) at a final concentration of 2μg/mL for 30min at 4 °C. Samples were stained according to the method mentioned above, using the following antibody: Penta-His AF488 (Qiagen, 35310), CTLA4 PE-Cy7. Data were acquired on LSRFortessa X-20 (Becton, Dickinson and Company) or CytoFLEX (Beckman Coulter).

### Mass cytometry

#### Preparation of cell suspensions

PBMCs from participants were washed in FACS buffer (phosphate buffered saline (PBS) supplemented with 0.5% bovine serum albumin) twice. Then, the number of cells was counted and the quality of samples for subsequent analysis should meet the following requirement: The number of cells should not be less than 3×10^6^ and the viability rate should be higher than 85%.

#### Preparation of antibodies

For mass cytometry analysis, purified antibodies were obtained from Biolegend, eBioscience, BioXcell, R&D systems and BD Biosciences, and clones were listed in **Supplementary Table 4**. Antibody labeling with the indicated metal tag was performed using the MaxPAR antibody Labelling kit (Fluidigm) according to the manufacturer’s instruction.

#### Mass cytometry staining, data acquisition

Cells were stained with 250nM cisplatin (Fluidigm) for 5min on ice to exclude dead cells, and then incubated in Fc receptor blocking solution before stained with surface antibodies cocktail for 30min on ice. Cells were washed and resuspend with deionized water, adding into 20% EQ beads (Fluidigm), acquired on mass cytometer (Helios, Fluidigm).

#### Mass cytometry data analysis

Data of each sample were normalized using bead normalization method (56). Then, debris, dead cells, doublets and non-immune cells (CD45^-^) were excluded through manually gating on FlowJo Software. Marker expression was arcsinh transformed, and X-shift clustering algorithm was applied to identify immune subsets. Raw metal ion counts from CyTOF acquisition were first normalized, then arcsinh-transformed with a cofactor of 5 to compress the wide dynamic range of signal intensities and reduce skewing of high-expression populations. t-Distributed Stochastic Neighbor Embedding (t-SNE) dimension reduction were performed to visualize cells.

### Statistical analysis

No data points were excluded from the analyses.

Data were presented at means ± SD, Median ± IQR. Pairwise comparisons were conducted applying Student’s t-test. For comparison of data in more than two independent groups, analysis of variance (ANOVA) was adopted with Turkey’s multiple comparisons if data were normally distributed, otherwise nonparametric Kruskal-Wallis test was used with Dunn’s multiple comparisons. The data were analyzed in GraphPad Prism 10. P < 0.05 was considered significant. *P < 0.05; **P < 0.01; ***P < 0.001; ****P < 0.0001; ns, not significant.

## Data Availability

The raw data supporting the conclusions of this article will be made available by the authors, without undue reservation.
