# Supplementary material for: Novel pathogenic CTLA4 missense variant associated with autoimmune enteropathy and systemic immune dysregulation
Source: Front Immunol. 2026 Jul 27;17:1855020. doi: 10.3389/fimmu.2026.1855020 (PMC13453822; doi:10.3389/fimmu.2026.1855020)
Supplement: Supplementary file 1 [file DataSheet1.docx]

**Supplementary material**

**Novel Pathogenic *CTLA4* Missense Variant Associated with Autoimmune Enteropathy and Systemic Immune Dysregulation**

**Supplemental Figure 1. The CTLA4 p.Phe56Cys (F56C) variant impairs CTLA4 protein expression and CD86-binding capacity**. (A) Flow cytometric analysis of CTLA4 surface expression and CD86-binding capacity in HEK293T cells transfected with plasmids encoding wild-type CTLA4 (F56), the CTLA4 p.Phe56Cys variant (C56), or an empty vector (EV); (B) Quantification of CD86-binding proportions, shown as percentage of CTLA4+CD86+ cells among all live HEK293T cells.

**Supplemental Figure 2. The CTLA4 p.Phe56Cys (F56C) variant is associated with an aberrant peripheral immune landscape.** (A) tSNE plots of PBMCs from the CTLA4 p.Phe56Cys variant patient, his nephew (an asymptomatic carrier of the same variant), and two CTLA4-wild-type healthy controls. Cells are colored according to cluster identity. (B) Heatmap showing marker expression profiles of the PBMC clusters identified in (A). Each row represents the median arcsinh-transformed intensities in each cluster. (C) tSNE plots of CD8+ T cells from the CTLA4 p.Phe56Cys patient, asymptomatic nephew, and healthy controls, with cells colored by cluster identity. (D) Heatmap showing marker expression profiles of the CD8+ T cell clusters identified in (C). (E) Quantification of CD8+ T cell cluster proportions in the three groups (corresponding to (C)). (F-G) CTLA4 expression levels (quantified by median arcsine-transformed intensities) across the major cell types identified in Figure 4(A) (showing in F) and the CD4+ T cell clusters identified in Figure 4(D) (showing in G).

**Supplemental table 1. Serum levels of immunoglobulins from the patient.**

| Immunoglobulins | Result | Unit | Reference interval |
| --- | --- | --- | --- |
| IgA | **0.45** | g/L | 1.45-3.45 |
| IgG | **8.25** | g/L | 10.13-15.13 |
| IgM | 2.07 | g/L | 0.92-2.04 |

**Supplemental table 2. Absolute counting of lymphocyte subpopulations from the patient.**

| Subpopulation | Result | Unit | Reference interval |
| --- | --- | --- | --- |
| CD45+ lymphocyte# | 1250.28 | /ul | 1142.00-3149.00 |
| T lymphocyte (CD3+) % | 85.45 | % | 49.10-83.60 |
| T lymphocyte (CD3+) # | 1068.36 | /ul | 876.80-2310.00 |
| B lymphocyte (CD19+) % | **5.47** | % | 6.50-19.35 |
| B lymphocyte (CD19+) # | **68.45** | /ul | 107.00-545.10 |
| T helper lymphocyte (CD3+CD4+) % | 50.44 | % | 23.20-52.20 |
| T helper lymphocyte (CD3+CD4+) # | 630.67 | /ul | 411.00-1228.88 |
| Cytotoxic T lymphocyte (CD3+CD8+) % | 32.23 | % | 15.08-39.62 |
| Cytotoxic T lymphocyte (CD3+CD8+) # | 402.95 | /ul | 258.90-958.70 |
| Natural killer cell % | 8.09 | % | 6.37-34.83 |
| Natural killer cell # | 101.12 | /ul | 109.80-780.40 |
| CD3+CD4+/CD3+CD8+ ratio | 1.57 |  | 0.60-2.88 |

**Supplemental table 3. Serum cytokine levels from the patient.**

| Cytokines | Result | Unit | Reference interval |
| --- | --- | --- | --- |
| IL-2 | 1.91 | pg/mL | 0.00-5.71 |
| IL-4 | 1.76 | pg/mL | 0.00-2.80 |
| IL-6 | **9.11** | pg/mL | 0.00-5.30 |
| IL-10 | **11.16** | pg/mL | 0.00-4.91 |
| Tumor necrosis factor | 1.24 | pg/mL | 0.00-4.60 |
| Interferon-gamma | 1.19 | pg/mL | 0.00-7.42 |
| IL-17 | 11.61 | pg/mL | 0.00-20.60 |

**Supplemental table 4. CyTOF panel used for evaluation of PBMCs from CTLA4 mutants and healthy controls.**

| Antigen | Clone | Mass& Metal label | Source | Cat# |
| --- | --- | --- | --- | --- |
| CD45 | HI30 | 89Y | Biolegend | 304002 |
| CD3 | UCHT1 | 115In | Biolegend | 300443 |
| CD62L | DREG-56 | 141Pr | Biolegend | 304854 |
| TCR γ/δ | 5A6.E9 | 142Nd | PLT | 100P001A |
| IgD | IA6-2 | 143Nd | Biolegend | 348202 |
| CD14 | 26ic | 144Nd | Biolegend | 301862 |
| CD20 | 2H7 | 145Nd | Biolegend | 302302 |
| CD38 | HIT2 | 146Nd | Biolegend | 303502 |
| CD66b | G10F5 | 147Sm | Biolegend | 305102 |
| CD185 (CXCR5) | RF8B2 | 148Nd | BD | 552032 |
| CD25 (IL-2Rα) | 24212 | 149Sm | RD | MAB1020 |
| CD56 | NCAM16.2 | 150Nd | BD | 559043 |
| CD80 (B7-1) | 2D10.4 | 151Eu | eBioscience | 14-0809-82 |
| CD11c | Bu15 | 152Sm | Biolegend | 337202 |
| CD196 (CCR6) | G034E3 | 154Sm | Biolegend | 353402 |
| CD123 (IL-3Rα) | 6H6 | 155Gd | Biolegend | 306002 |
| CD86 | FUN-1 | 156Gd | BD | 555655 |
| CD183 (CXCR3) | G025H7 | 157Gd | Biolegend | 353750 |
| CD19 | HIB19 | 158Gd | Biolegend | 302268 |
| CD45RO | UCHL1 | 159Tb | Biolegend | 304202 |
| CD194 (CCR4) | L291H4 | 160Gd | Biolegend | 359402 |
| CD152 (CTLA-4) | BN13 | 161Dy | Bioxcell | BE0190 |
| CD10 | HI10a | 162Dy | Biolegend | 312202 |
| CD161 | HP-3G10 | 163Dy | Biolegend | 339902 |
| CD57 | HNK-1 | 164Dy | Biolegend | 359602 |
| CD294 (CRTH2) | BM16 | 165Ho | Biolegend | 350102 |
| CD27 | O323 | 166Er | Biolegend | 302802 |
| CD197 (CCR7) | G043H7 | 167Er | Biolegend | 353256 |
| IgM | MHM-88 | 168Er | Biolegend | 314502 |
| CD16 | 3G8 | 169Tm | Biolegend | 302057 |
| CD127 (IL-7Rα) | A019D5 | 170Er | Biolegend | 351302 |
| CD28 | CD28.2 | 171Yb | Bioxcell | BE0291 |
| IgG | G18-145 | 172Yb | BD | 555784 |
| CD278 (ICOS) | C398.4A | 173Yb | Biolegend | 313502 |
| CD279 (PD-1) | EH12.2H7 | 174Yb | Biolegend | 329926 |
| CD45RA | HI100 | 195Pt | Biolegend | 304143 |
| CD8 | HCD8-06 | 196Pt | ZXBIO | 110010402 |
| CD4 | RPA-T4 | 197Au | Biolegend | 300541 |
| HLA-DR | L243 | 198Pt | Biolegend | 307648 |
| CD11b | M1/70 | 209Bi | Biolegend | 101202 |
